# Full-dimensional dynamic convolution and progressive learning strategy for strawberry recognition based on YOLOv8

**DOI:** 10.3389/fpls.2025.1541365

**Published:** 2025-03-31

**Authors:** Liping Bai, Chenglei Xia, Fei Liu, Xing Yang, Tai Zhang

**Affiliations:** Macau Institute of Systems Engineering and Collaborative Laboratory for Intelligent Science and Systems, Faculty of Innovation Engineering, Macau University of Science and Technology, Macau, Macao SAR, China

**Keywords:** strawberries recognition, target detection, improved YOLOv8, EfficientNetv2, ODConv, Wise-IoU

## Abstract

The growth of strawberries is influenced by environmental diversity and spatial dispersion, which present significant challenges for accurate identification and real-time image processing in complex environments. This paper addresses these challenges by proposing an advanced recognition model based on YOLOv8, tailored for strawberry identification. In this study, we enhanced the YOLOv8 architecture by replacing the traditional backbone with an EfficientNetV2 feature extraction network and using ODConv instead of the standard convolution. The loss function was modified with a dynamic nonmonotonic focusing mechanism, and WiseIoU was introduced to replace the traditional CIoU. The experimental results showed that the proposed model outperformed the original YOLOv8 regarding mAP50, precision, and recall, with improvements of 16.91%, 14.92%, and 8.4%, respectively. Additionally, the model's lightness increased by 15.67%. The proposed model demonstrated superior accuracy in identifying strawberries of different ripeness levels. The improvements in the proposed model indicate its effectiveness in strawberry recognition tasks, providing more accurate results in varying environmental conditions. The lightweight nature of the model makes it suitable for deployment on picking robots, enhancing its practical applicability for real-time processing in agricultural settings.

## 1 Introduction

The strawberry cultivation and sales market is continuously growing, and the United States is one of the largest producers with an annual output of approximately 1.5 million tons, accounting for one-third of the total global production. Spain and Poland are the major producers in Europe, with annual outputs of 600,000 and 500,000 tons, respectively (Zhang et al., 2024b). China is the world’s largest strawberry producer, with a dedicated cultivation area exceeding 100,000 ha, and an annual output of 3 million tons (Lei et al., 2021). Based on data from 2023, China’s total strawberry production is expected to reach 2.2 million tons with a cultivation area of approximately 120,000 ha. The main growing provinces are Shandong, Sichuan, and Jiangsu, and their market size is projected to reach 45 billion yuan, with expectations exceeding 50 billion yuan by 2025.

Strawberry recognition faces multiple challenges in terms of object detection. First, strawberries are typically small and often heavily overlapped or occluded by other plant parts such as leaves and vines, making their detection difficult. Particularly in complex backgrounds, strawberry detection requires high precision and robustness to effectively distinguish strawberries from the background. Second, the appearance of strawberries changes significantly depending on factors such as ripeness, variety, and lighting, thereby increasing the detection complexity. Strawberries with different ripeness levels have noticeable differences in color and shape, transitioning from bright red to green, which raises the demand for classification and recognition. Strawberry detection systems are typically deployed in agricultural automation equipment. Therefore, strawberry recognition systems must address multiple challenges, including small-object detection, complex backgrounds, appearance variations, and device adaptability. It is also vital to synergize robotic technology to implement robotic harvesting of target fruits (Jin, 2020; Jichao and Fengzhi, 2021).

Scholars have proposed various methodologies for recognizing targeted fruits and vegetables. These methods include traditional image-technology-based approaches, such as color segmentation and shape matching. Alternatively, machine vision technology-based recognition methods such as template-based matching and pattern-based recognition have also been employed. Kurtulmus et al. (2011) use three different proportions of moving subwindows to scan the entire image to locate the target fruit. Each subwindow performs three classifications using intensity components, saturation components, and circular Gabor textures, and finally determines the result of the subwindow classifier. The algorithm achieved a detection success rate of 75.3%. Restrepo-Arias et al. (2022) introduce a target recognition method based on texture features, which avoids classification biases caused by leaf shape and achieves a precision of 96.31%. However, this process is affected by inconsistent illumination.

With the rapid development of neural network technology, researchers have been exploring the use of neural networks for target detection. Garcés Cadena et al. (2023) and Meng et al. (2023) propose a spatio-temporal convolutional neural network in combination with a Transformer fusion regional convolutional neural network for detecting pineapple fruit. This method achieved a recognition precision of 92.54%, with an average reasoning time of 0.163s. Zhang et al. (2024a) ResNeXt-50 is used as the backbone network to optimize the anchor boxes in the RPN layer, adapting to the complex morphology of pepper branches. The improved model achieved recognition accuracies of 92.2%, 96.3%, and 85.6% for upright, centripetal, and competing branches, respectively. Jia et al. (2020) combined ResNet and DenseNet to significantly reduce the number of input parameters. Finally, the mask generated by the FCN is used to obtain the region where the apples are located. This method achieved an accuracy of 97.31% and recall rate of 95.70%. The green apple detection method proposed by Ji et al. (2025) is based on an improved DETR model, which combines a multidimensional feature extraction network and transformer module to significantly enhance detection accuracy and efficiency. The transformer module employs a multiscale deformable attention mechanism, optimizing the model’s convergence speed and computational efficiency. However, the model has high computational complexity and requires substantial computational resources, especially on embedded platforms, which may affect the real-time performance. Fujinaga (2024) proposed a deep-learning-based method for a multifunctional agricultural robot designed for strawberry harvesting and inflorescence pruning. The method utilizes the semantic segmentation model DeepLabV3+ to identify components, such as strawberries, inflorescences, and sepals, and extract their relevant attributes. Based on the extracted attributes, the robot detects the cutting points for fruit harvesting and inflorescence pruning, while avoiding non-working areas that may potentially damage the plants. The method demonstrated high accuracy with F-measures of 0.93 for fruit detection and 0.86 for pruning cutting points, and it performed stably under various lighting conditions. However, when multiple fruits are in close proximity, especially along the vertical axis, semantic segmentation treats them as a single large region, resulting in an inability to accurately separate and identify them as individual fruits. Additionally, when fruits are tilted, the sepals, which should be positioned above the fruit, may no longer align accurately, leading to errors in cutting-point localization. Furthermore, this method determines fruit maturity based on a specific threshold, with only two categories: mature and immature.

The YOLO algorithm is popular owing to its parameter-light and deployable nature. Chen et al. (2023) proposes a YOLO-COF lightweight occlusion target detection method for identifying camellia fruit. The YOLOv5s model utilizes the K-means clustering algorithm to select and automatically filter target datasets, and the attention mechanism enhances the feature extraction of obscured targets. The experimental data indicate an mAP value of 94.10% and a model size of 27.1 MB. Chen et al. (2023) propose an improved apple picking method that combines the EF-YOLOV5s object detection network and an enhanced DBSCAN-based picking order planning. EF-YOLOV5s significantly improves apple recognition accuracy in complex environments by introducing the EfficientFormer structure and Soft-NMS algorithm, but it increases the model’s complexity, particularly in resource-constrained scenarios. The modified DBSCAN algorithm automatically groups apples into different picking clusters and plans the picking sequence using Gaussian weighting and saliency levels, thereby improving both the efficiency and success rate. However, the effectiveness of the DBSCAN algorithm depends on the choice of the parameters. If the density distribution of the clusters is complex, the automatically selected parameters may not fully adapt to all scenarios. Additionally, this method focuses solely on apple detection and picking-order planning without specifically addressing the classification of apple maturity.

However, these algorithms require significant computing resources, which leads to performance degradation during deployment. Consequently, they are not suitable for low-resource devices. To address this issue, we propose a new YOLOv8 object detection model by replacing the original backbone structure with the EfficientNetV2 network. To maintain or enhance recognition precision while keeping the model lightweight, we optimized the neck structure and introduced ODConv, employing a parallel strategy to learn attention values within the convolution kernel across four dimensions, thus enhancing the information fusion capabilities. In addition, we incorporated Wise-IoU to improve the model’s generalization ability. This aids in identifying small targets in complex environments and enhances categorization precision. Finally, we applied the new model to a strawberry dataset to validate these methods.

The remainder of this study is organized as follows. *Section 2* presents the data collection and processing. *Section 3* introduces the proposed method and *Section 4* presents the experimental results. Finally, *Section 5* provides an overview of the conclusions and recommendations for future research.

## 2 Materials and methods

### 2.1 Experimental data acquisition

Strawberry images were collected at the Xiangyuan Strawberry Garden in Xiangzhou District, Zhuhai City, Guangdong Province. The collection was conducted under naturally lit, clouded conditions to replicate the most common environmental conditions during the picking season. The equipment utilized for image capture was a HUAWEI Mate30E Pro with a 40-megapixel ultra-sensitive camera, and all photographs were saved in JPG format.

**Figure 1:**
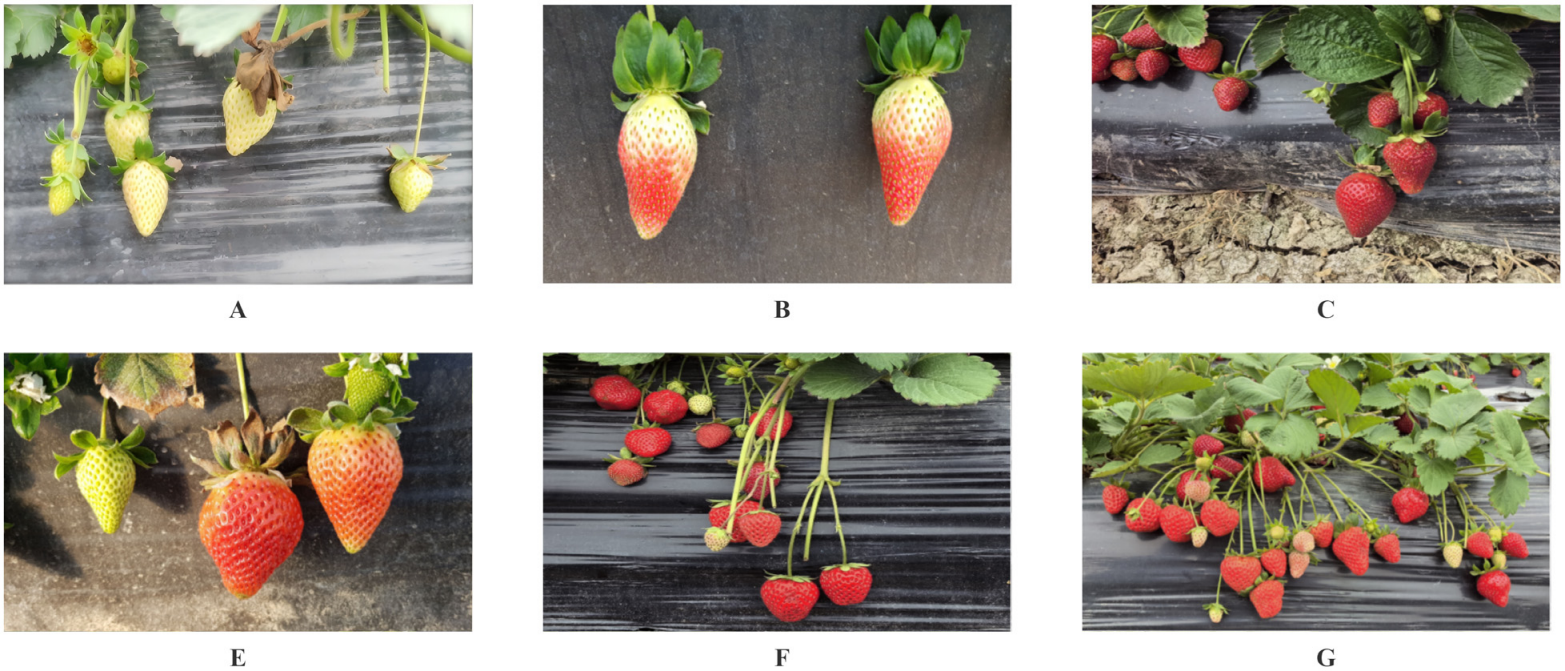
Photographs taken in different environments. **(A)** Low ripeness strawberries; **(B)** Medium ripe strawberries; **(C)** High ripeness strawberries; **(D)** Strawberries in direct sunlight; **(E)** A close-up shot of strawberries; **(F)** Strawberry shot from a distance; **(G)** Strawberry shot from a longer distance.

To align with real-world scenarios of robotic harvesting, we mounted the camera at a height and angle consistent with that of the camera installed on the robot. We amass 1,680 original strawberry images depicting various conditions, including overlapping, backlighting, and occlusion (**Figure 1**).

The harvesting period was between February and May; therefore, we chose it as a relevant subject for our research.

### 2.2 Generate data set

**Figure 2:**
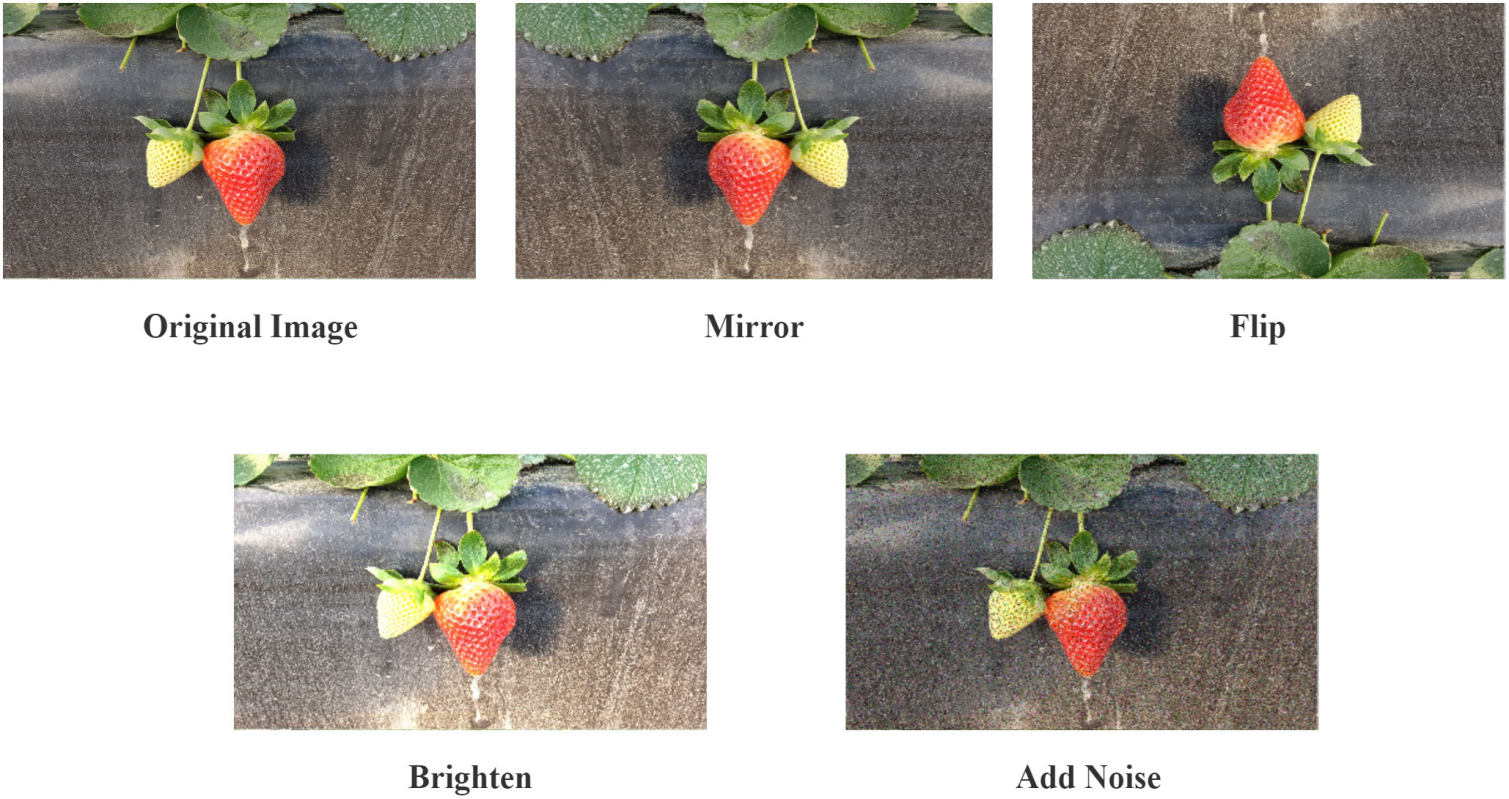
Image data augmentation methods.

As deep learning models require extensive data for training, the quality of the dataset has a significant impact on model performance. Therefore, it is necessary to use various data augmentation techniques to enrich existing data. In this study, we used various image enhancement methods, including rotation, translation, and brightness adjustment (**Figure 2**). All the images were resized to a fixed size of 640 × 640 pixels. Through this data augmentation, we significantly expanded our dataset and amass 3,500 photographs.

**Figure 3:**
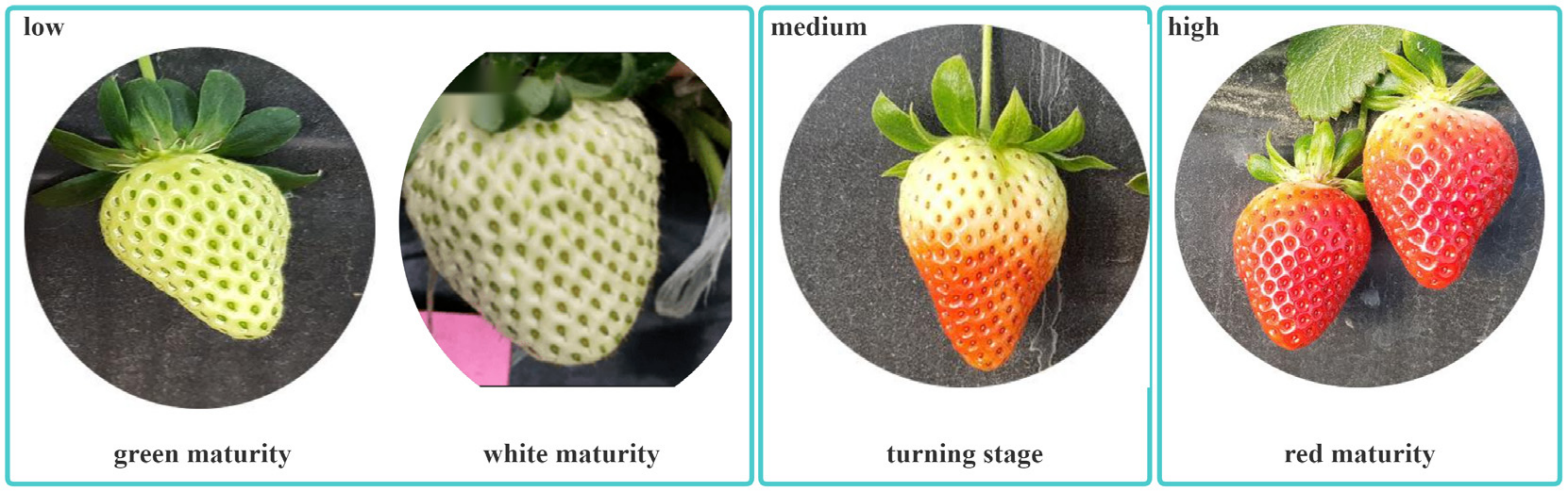
Classification of different strawberry maturity levels.

Strawberry ripeness can be divided into four stages: green, white, color change, and red ripening. To ensure the local supply of strawberries and to transport the harvested strawberries to relatively distant areas for sale, fruit farmers must harvest not only completely ripe red strawberries but also strawberries in the color-changing stage. Therefore, we divided strawberry maturity into three phases: low, medium, and high (**Figure 3**). Local transportation is best for high maturity, which is also known as red ripeness. Long-distance shipping is suitable for medium ripeness, which corresponds to the color-changing stage. A low maturity indicates an immature stage that requires additional growth.

**Figure 4:**
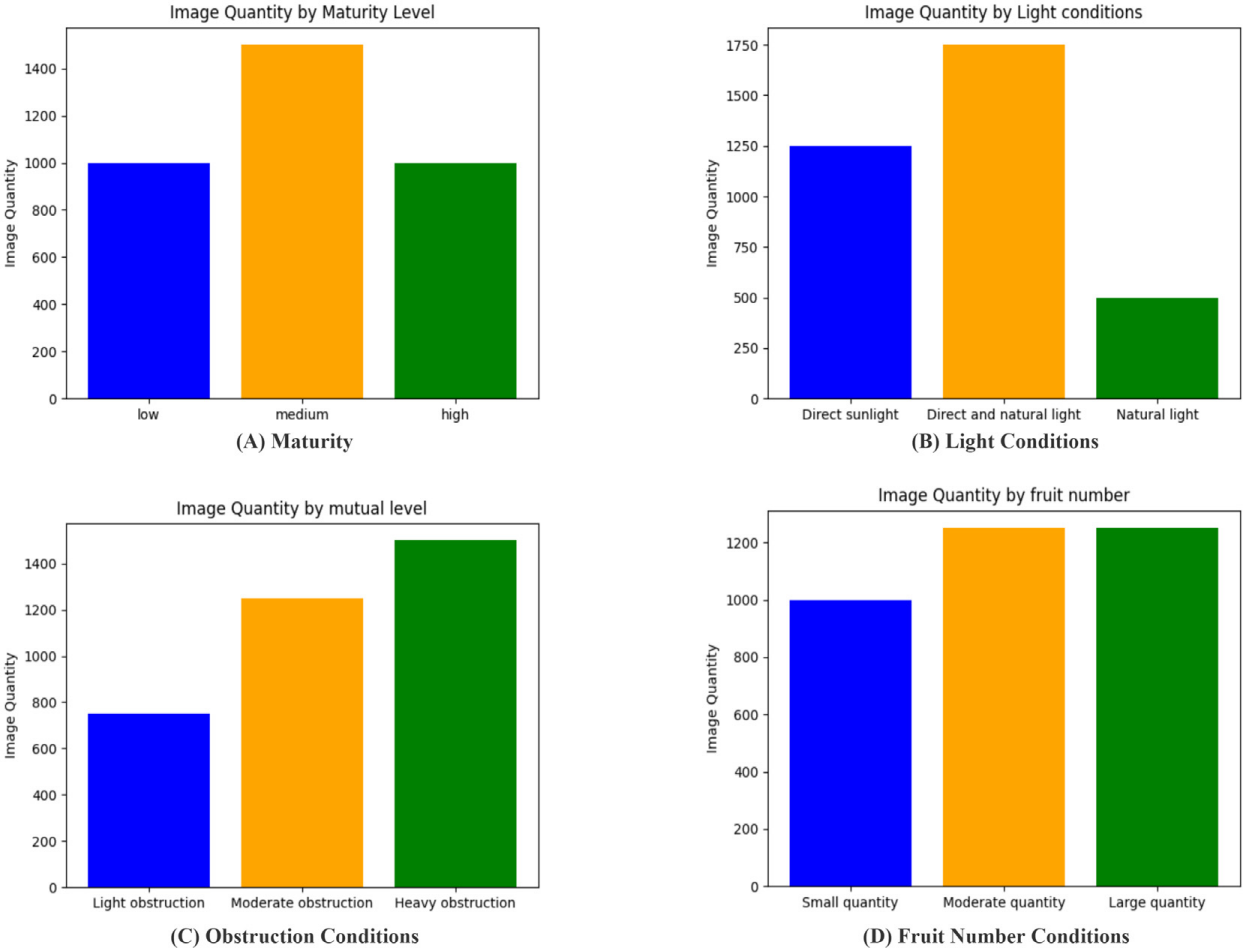
The number of images of different types. **(A)** Maturity Conditions; **(B)** Light Conditions; **(C)** Obstruction Conditions; **(D)** Fruit Number Conditions.

We statistically determined and set the number of images under different conditions based on the difficulty of training (as shown in **Figure 4**). This study separately counted the number of images for four classification methods: maturity, light intensity, degree of occlusion, and number of strawberries in a single image. In terms of maturity, images with medium maturity were more difficult to identify; therefore, their number was higher than that of both the low- and high-maturity images. Regarding light intensity, images under natural light were easier to identify, resulting in the fewest images, whereas images with both direct sunlight and automatic light were the most numerous. The number of severely occluded images was the highest for the degree of occlusion. Finally, in terms of the number of strawberries per image, the number of images with many or moderate strawberries was equal. In addition, the training, validation, and test sets were split in an 8:1:1 ratio, meaning the training, validation, and test sets contained 2,800, 250, and 250 images, respectively.

Labeling image annotation software was used to input all prepared image data and establish three maturity categories: low, medium, and high. The results were obtained after annotation. The.txt files were used as datasets for training models in subsequent activities.

## 3 Object detection model

### 3.1 YOLOv8 network

The YOLO algorithm is a popular object-detection method introduced by Redmon et al., in 2016. It extracts features from an entire image and predicts object locations and categories within a single neural network, combining object detection and image classification tasks. YOLO has achieved significant improvements in its computational efficiency and detection capabilities (Liu et al., 2018).

Compared with the previously proposed YOLO series algorithms, the YOLOv8 algorithm improves the backbone network and neck by incorporating the extension-elan idea proposed by the YOLOv7 network (Wang et al., 2023) and enhancing the C3 module in YOLOv5 (Yang et al., 2020) into the C2f module with more abundant gradient flow. It can adjust the number of different channels for different-scale models. There were significant changes in the head. Compared with YOLOv5, the decoupling head structure is replaced, the classification and detection heads are separated, and an anchor-free concept is introduced to replace the original anchor-based one. In terms of data enhancement, the operation of turning off the mosaic enhancement in the last 10 generations of training in the YOLOX (Ge, 2021) model is introduced, which helps to improve the precision of the model.

In the loss function, YOLOv8 differs from previous YOLO models by using one-hot encoding for classification loss to determine the presence of objects instead of object loss. Both the v5 and v8 models use binary cross entropy (BCE) as the criterion for classification loss (Wu et al., 2022). The expression for the BCE loss is shown in Equation 1:


(1) $$L=\frac{1}{N}\sum_{i}-[y_{i}\text{ log }P_{i}+(1-y_{i})\text{ log }(1-P_{i})]$$

The other loss is the regression loss. In the regression task, the degree of regression was measured using the ratio of the target and prediction frames. The IoU used by v8 was CIoU (Zheng et al., 2020), and the aspect ratio criterion was added to the original intersection ratio loss. The expression formula for this criterion is shown in Equation 2:


(2) $$L_{CIoU}=1-IoU+\frac{\rho^{2}(b,b^{gt})}{c^{2}}+\alpha v$$

Due to the introduction of Anchor-Free in the v8 model and to improve generalization, the DFL loss (Li et al., 2020) has been increased. The expression for the DFL loss is shown in Equation 3:


(3) $$DFL(S_{i},S_{i+1})=-((y_{i+1}-y)\text{ log }S_{i}+(y-y_{i})\text{ log }S_{i+1})$$

### 3.2 Improved YOLOv8 recognition model

Because YOLOv8 detects small targets, a lightweight processing approach is necessary for the recognition model to be deployed and to communicate with robotic arms and autonomous vehicles. Therefore, we propose an improved yolov8 model. We replaced the original backbone structure with the EfficientNetV2 feature extraction network and performed lightweight processing on the model. We replaced the original standard convolution operation with the ODConv convolution operation in the head part. The model’s recognition efficiency in complex environments is improved by using a multidimensional attention mechanism that learns complementary attention along the four dimensions of the kernel space via a parallel strategy. The original CIoU was replaced with Wise-IoU in the loss function using a dynamic non-monotonic focusing mechanism.

#### 3.2.1 Improvement of model structure

**Figure 5:**
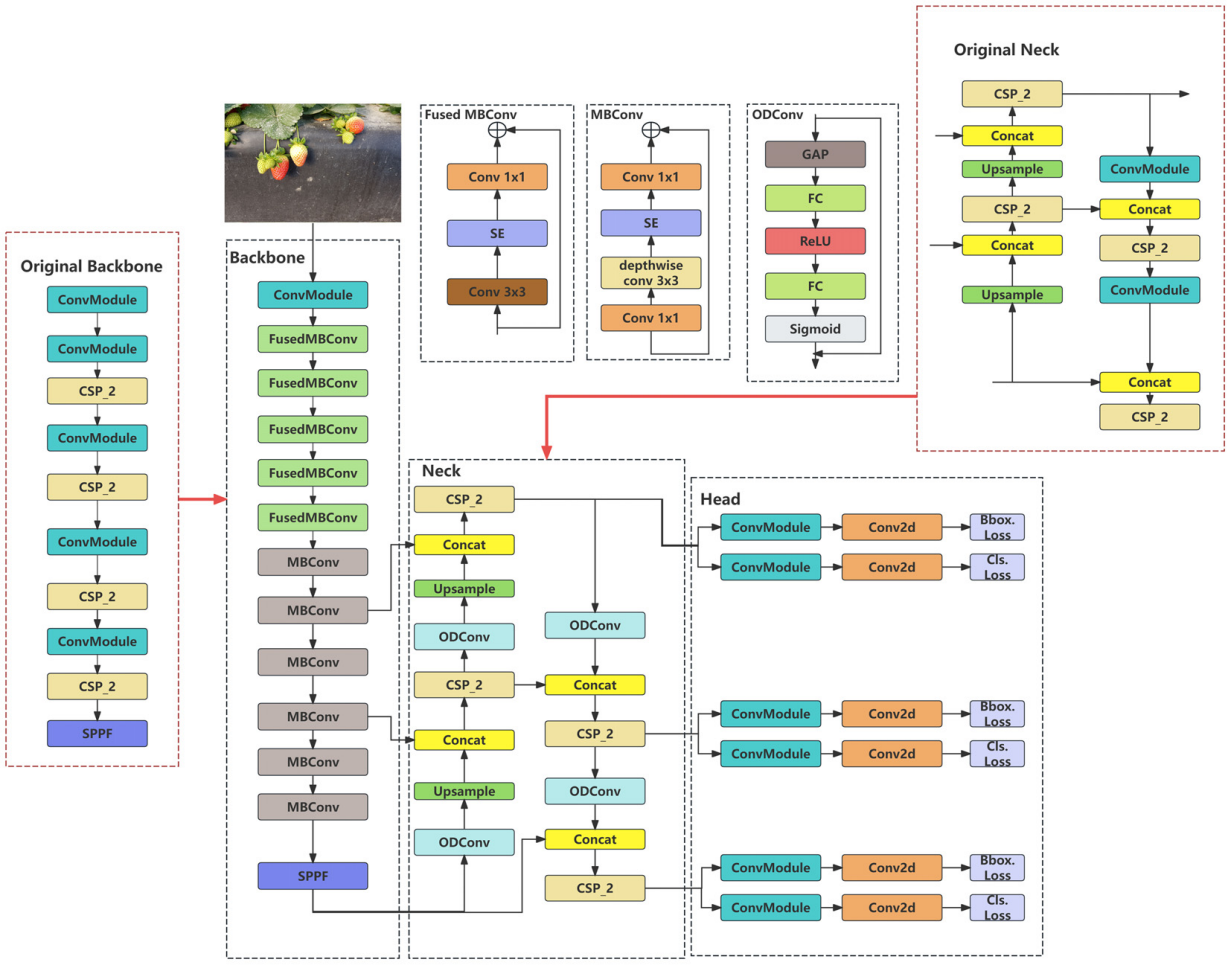
Improved YOLOv8 network structure.

As the recognition model must be deployed in practical applications, it is necessary to improve the existing model by minimizing the number of parameters without reducing its precision or performance. After conducting several experiments, we proposed an improved YOLOv8 model, as shown in **Figure 5**.

First, we achieved model simplification by modifying the network architecture. EfficientNetV2 is used to replace the original network in the backbone network, such that the parameter count is decreased and the model lighter.

However, this simplified model may lead to potential reductions in the recognition precision, recall rates, and other performance metrics. To address this issue, two ODConv operations were applied in the up-sampling section of the neck stage, and the original Conv structure in the down-sampling section was replaced with ODConv. The ODConv model utilizes a multidimensional attention mechanism in parallel strategies to provide flexible attention to learning in four dimensions of the convolution kernel space. This improves the model’s performance.

To ensure robustness when encountering objects of different sizes and obstructed objects during the recognition process, it is necessary to use an IoU with solid robustness. The traditional IoU is more sensitive to the position and size of the label box and cannot adapt to dense or sparse objects. Therefore, the model adopts wise-IoU, which not only has good detection accuracy, but is also better suited for overlapping and sparse targets.

#### 3.2.2 EfficientV2 backbone architecture

Owing to the use of deep convolution in the shallow layers of the network, the EfficientNetV1 model (Tan, 2019) experiences a slowdown in training speed when the size of the training images increases significantly. In addition, the depth and width of each model stage were equally enlarged, further contributing to the slow running speed. However, each stage contributes differently to the training speed and number of parameters of the network. Therefore, it is not reasonable to directly use the strategy of equal scaling.

**Figure 6:**
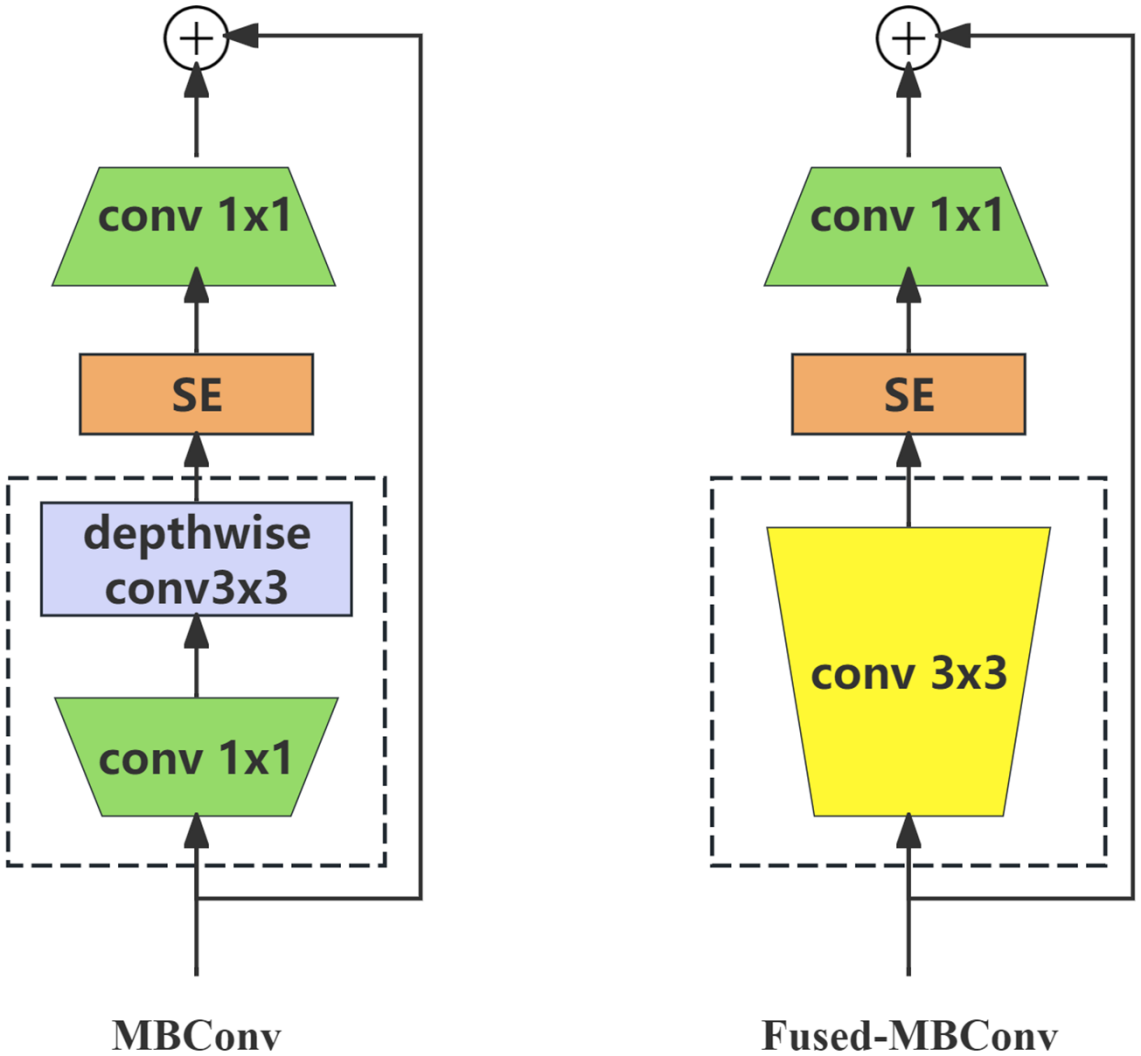
The structure of MBConv and fused-MBConv.

To address these issues, Tan and Le (2021) propose the EfficientNetV2 model. The original model’s depth wise convolutions are not able to fully utilize the accelerators available in the current hardware, leading to slower practical application compared to the theoretical performance. A new fused-MBConv architecture (shown in **Figure 6**) is introduced to enhance the computational efficiency and leverage existing hardware capabilities, thereby achieving faster real-world performance.

**Figure 7:**
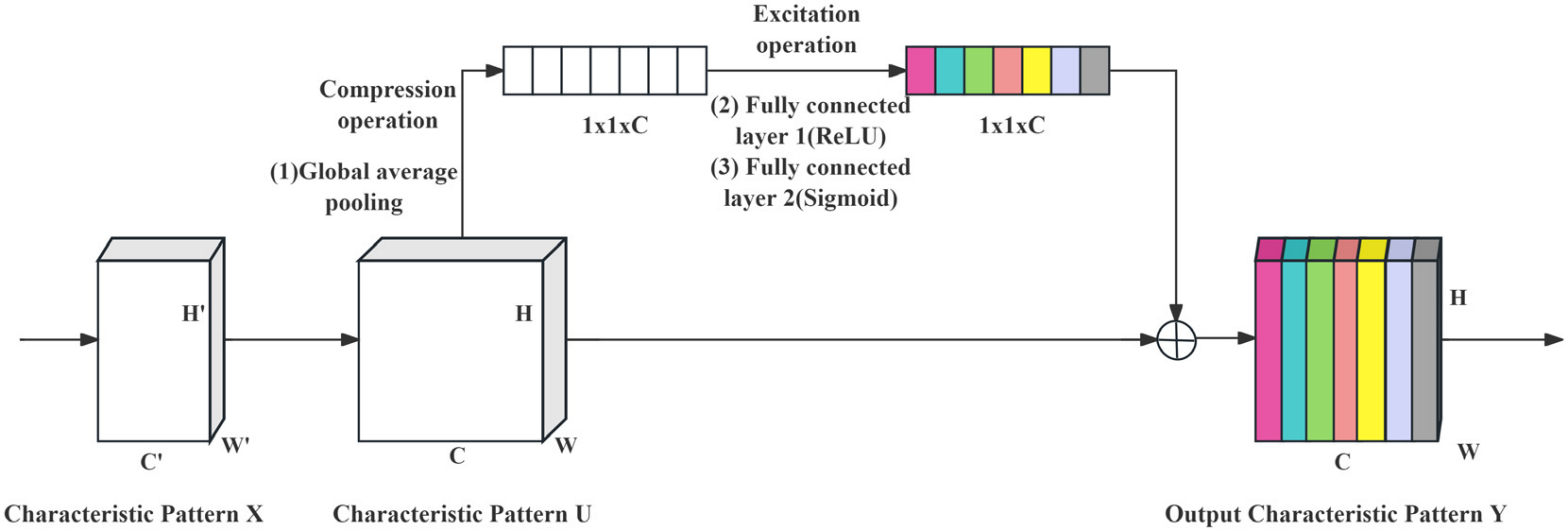
SE channel attention mechanism diagram.

The structure was updated to combine the 1 × 1 and deep convolutions of the MBConv module into a 3 × 3 convolution. Additionally, both structures incorporate the SE channel attention mechanism (Hu et al., 2018), which adjusts the weight value of each channel on the short-cut branch to improve or depress the importance of different channels during the training process. A schematic of this process is shown in **Figure 7**.

The SE module uses global average pooling to compress the two-dimensional features of each feature map into a single value. This stage involves a structure with two fully connected layers, where the first layer compresses the channels and reduces their number to decrease the computational load. After activation by the ReLU activation function, the dimension was restored to the input dimension at the beginning of the branch following the second fully connected layer. Finally, the weight coefficient is activated by the sigmoid activation function.

To achieve the correct combination of the two modules, we used a NAS search to replace the first four MBConvs in the network with fused-MBConv modules. This combines the precision, efficiency, and training speed of the modern accelerators.

During the initial training phase, it is essential to increase the image size using smaller images and weak regularization. It also makes it more challenging to add regularization learning. Thus, the network enhanced progressive learning and introduced adaptive regularization, as shown in **Algorithm 1**. The training process is divided into M stages. During the first M-1 stages, the model was trained using the image size and regularization amplitude, and then linear interpolation was used to determine the value of each stage. The final stage, M, uses the target image size and regularization.

Algorithm 1 **Adaptive regularization progressive learning**

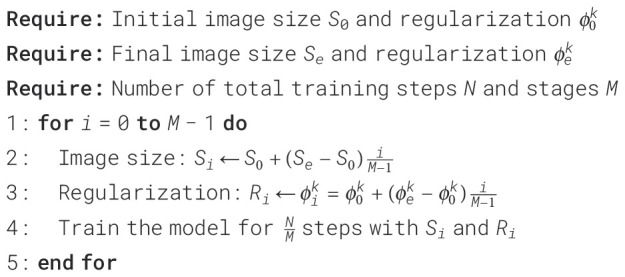

**Table 1** lists the proposed backbone architecture. The first stage consists of standard 3×3 convolutions. Stages 2–6 stack fused-MBConv structures, stages 7–12 are composed of MBConv structures, and the final stage uses the SPPF structure.

**Table 1:** EfficientNetV2 network architecture.

Stage	Operator	Stride	Channels	Expansion
1	Conv 3 × 3	2	24	2
2	Fused-MBConv, k3 × 3	1	24	1
3	Fused-MBConv, k3 × 3	2	48	4
4	Fused-MBConv4, k3 × 3	1	48	4
5	Fused-MBConv, k3 × 3	2	64	4
6	Fused-MBConv4, k3 × 3	1	64	4
7	MBConv, k3 × 3, SE0.25	2	128	4
8	MBConv, k3 × 3, SE0.25	1	128	4
9	MBConv, k3 × 3, SE0.25	2	160	6
10	MBConv, k3 × 3, SE0.25	1	160	6
11	MBConv, k3 × 3, SE0.25	2	256	4
12	MBConv, k3 × 3, SE0.25	1	256	4
13	SPPF	–	1,024	–

#### 3.2.3 Omni-dimensional dynamic convolution

Conventional convolution utilizes a single static convolution kernel and is not affected by the input sample. However, the weight values depend on the input, leading to input dependency. CondConv, the pioneering work on dynamic convolution proposed by Yang et al. (2019), utilizes various convolution kernels for different inputs and weights multiple convolution kernels linearly. However, the weighted value depends on the input situation, thus making the dynamic convolution dependent on the input. Based on this model, Chen et al. (2020) improved the attention mechanism in DyConv and allocated the extracted attention to different convolution kernels using the SENet method.

While the previous models only considered the dynamic characteristics of convolutional cores for the number of convolutional cores, they ignored the space size of each convolutional kernel, the number of input channels, and the number of output channels. This limitation leads to lower performance of such convolutions in large networks. This study employs omni-dimensional dynamic convolution to address the limitations of previous models.

**Figure 8:**
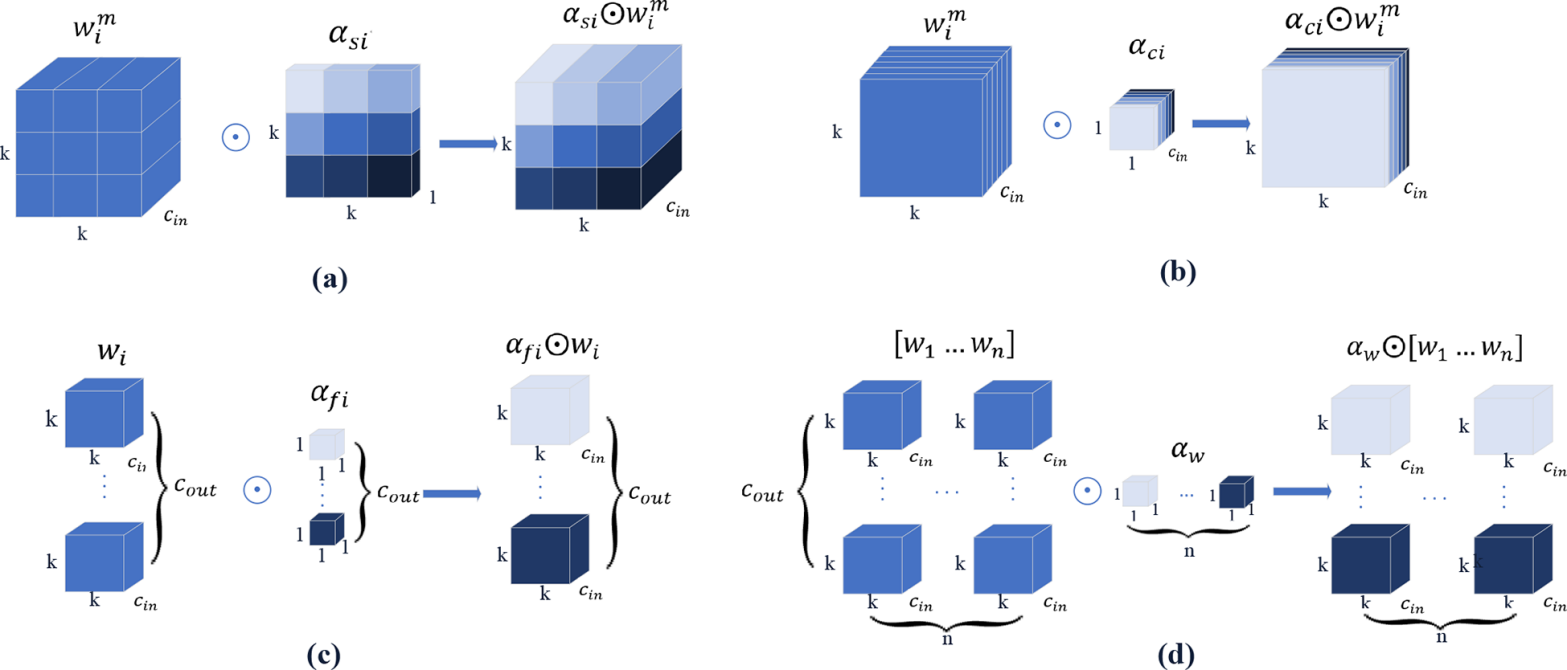
Four dimensions attention diagram.

ODConv uses multidimensional attention mechanisms and parallel strategies to learn convolution kernel attention across the four dimensions of the kernel space at any convolution layer. A four-dimensional diagram of the attention is shown in **Figure 8** (Li et al., 2022).

**Figures 8a–c** show that different attention values are assigned to the convolution parameters of the spatial position, input channel convolution filter, and output channel convolution filter. For a single convolution kernel, in **Figure 8d**, different values are assigned to n global convolution kernels.

Omni-Dimensional Dynamic Convolution improves the performance of convolutional neural networks in complex tasks by dynamically generating convolution kernels and processing multidimensional features. The calculation process for ODConv is as follows:

Given an input feature map *X* ∈ ℝ*^H^* ^×^ *^W^* ^×^ *^C^*, where *H* is the height, *W* is the width, and *C* is the number of channels, the generation of the convolution kernel *K* is dynamic, depending on the input features *X* and contextual information. The dynamic generation of the kernel *K* is shown in Equation 4:


(4) K=f(X,θ)

where *f*(·) is the generation function and *θ* represents the learnable parameters that determine the shape and size of the kernel. Based on the generated convolution kernel *K*, ODConv performs a standard convolution operation, but the kernel is dynamically adjusted for each input. Therefore, the calculation method for the output *Y* is shown in Equation 5:


(5) $$Y=\sum_{i=1}^{H}\sum_{j=1}^{W}K(i,j)\cdot X(i,j)$$

where *Y* is the output feature map, representing the result of applying the dynamically generated convolution kernel *K* to the input feature map *X*.

#### 3.2.4 Wise-IoU

Intersection ratio (IoU) denotes the ratio of intersection and union of two regions, as shown in Equation 6:


(6) $$L_{IoU}=1-\frac{W_{i}H_{i}}{wh+w_{gt}h_{gt}-W_{i}H_{i}}$$

The anchor frame is $\vec{B}=[xywh]$ , and the target frame is $\vec{B_{gt}}=[x_{gt}y_{gt}w_{gt}h_{gt}]$ . However, IoU (Jiang et al., 2018) has a flaw that when there is no overlap between bounding boxes, the gradient of back propagation disappears, and the width of the overlap area is not able to update at training time.

Because the calculation of CIoU in the original YOLOv8 model is relatively complex, the training stage requires a large amount of computing resources and the training set contains low-quality examples. If only the regression of the boundary box contains low-quality examples, it hinders the performance improvement of the entire model. Therefore, we use a dynamic nonmonotonic focusing mechanism combined with a gradient gain distribution strategy (Tong et al., 2023) to reduce the harmful gradient of low-quality examples while reducing the competition for high-quality anchors.

Because the conventional set measurement factors aggravate the penalty for low-quality examples and reduce the generalization ability of the model, we need to weaken the penalty of the set measurement. Therefore, distance attention was constructed using distance measurement, and WIoU v1 with a two-layer attention mechanism was obtained. The definition formula is as follows:


(7) $$L_{WIoUv1}=R_{WIoU}L_{IoU}$$

(8) $$R_{WIoU}=exp(\frac{{\left(x-x_{gt}\right)}^{2}+{\left(y-y_{gt}\right)}^{2}}{{\left(W_{g}^{2}+H_{g}^{2}\right)}^{*}})$$

In Equation 7, *R_WIoU_* ∈ [1*,e*] significantly increases the number b, that is, the ordinary mass anchor frame. For Equation 8, if there are two anchor frames with the same *IoU* for the target frame, the anchor frame *R_WIoU_* with a relatively far center distance is better than the other. The anchor frame *L_WIoUv_*_1_ is also significant; therefore, more attention will be paid to the anchor frame with a far center distance. Simultaneously, we introduce an outlier degree to define the mass of the anchor frame. The definition formula is as follows:


(9) $$\beta =\frac{L_{IoU}^{*}}{L_{IoU}^{-}}=\frac{L_{IoU}^{*}}{(1-M)L_{IoU}^{-}+M\times L_{IoU}^{*}}\in [0,+\infty )$$

A small outlier means a high-quality anchor frame; therefore, a small gradient gain is needed to make the boundary frame regression focus on the ordinary quality anchor frame, which results in the improvement of the generalization ability of models because the number of ordinary quality anchors is larger than that of high-quality and low-quality anchors. $L_{IoU}^{-}$ is the sliding average of momentum m. Since it is dynamic, the mass division criteria of the anchor frame are also dynamic, so the model makes a gradient gain allocation strategy conform to the current situation.

On this basis, *β* is used to construct the non-monotonic focusing coefficient and *r* is a scaling factor that ensures that the weighting of the loss function is proportional to the quality of the anchor boxes, as shown in Equation 10, thus adjusting the model learning based on the quality of the target boxes. Through *r*, the weighting of the loss function can be adjusted adaptively according to the quality of the boxes. High-quality boxes (i.e., those with smaller *β*) will have larger weights, making their loss contributions more significant for model updates. For lower-quality boxes (i.e., those with a larger *β*), the weighting is smaller, reducing their impact on training and preventing the model from overoptimizing low-quality target boxes.


(10) $$r=\frac{\beta }{\delta \alpha^{\beta -\delta }}$$

WIoUv3 is the version of the loss function based on the *β* and weighting mechanism, we have the formula WIoUv3 is shown in Equation 11:


(11) $$L_{WIoUv3}=rL_{WIoUv1}$$

### 3.3 Model evaluation index

The training utilized a V100 GPU with 32 G of video memory and an Intel Xeon Processor (Skylake, IBRS), and was trained on the Windows 11 operating system with an initial weight learning rate of 0.001, attenuation coefficient of 0.0005, and 300 iterations.

The experiment employed the mAP value, precision, and recall rate as evaluation indicators, with the precision and parameters being the most significant. Recognition was classified into three types: low, medium, and high. Among them, the formulas for the precision and recall are shown in Equation 12:


(12) $$Precision=\frac{TP}{TP+FP},Recall=\frac{TP}{TP+FN}$$

where TP represents a predicted box with an IoU greater than a specified threshold. FP represents a predicted box with an IoU less than or equal to the specified threshold. FN indicates a genuine target box without a TP.

The area under the PR curve is the Average Precision (AP), and mAP is the AP average of all categories. The parameters are introduced as evaluation indexes to display the model’s lightweight degree. Each number corresponds to the weight matrix in the convolution and full join operations used in the model, and is composed of multiple parameters. A smaller value indicated a less complex model.

The mAP50 is a commonly used evaluation metric to assess the model performance. It evaluates the accuracy of the model in detection tasks by calculating the average precision (AP) at an IoU threshold of 0.5. First, the AP value for each class is computed, which is the average precision obtained from the precision–recall curve at different confidence thresholds. The AP values of all classes were then averaged to obtain the final mAP50 score. As a standardized evaluation metric, the higher the mAP50 value, the better the model’s performance in terms of detection accuracy.

## 4 Analysis of experiment results

### 4.1 Experimental results

**Figure 9:**
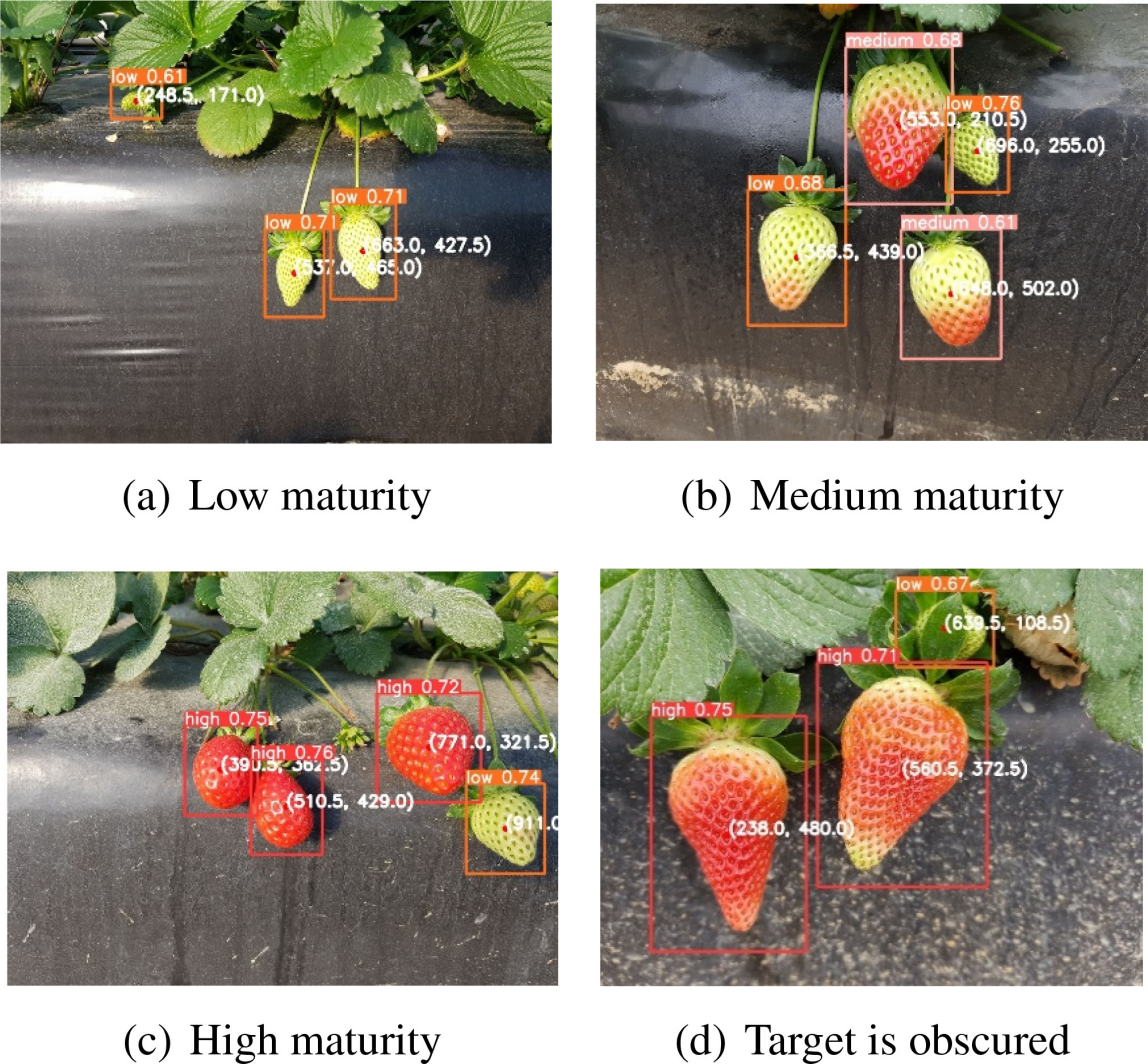
Detection in different scenarios. **(a)** Low maturity; **(b)** Medium maturity; **(c)** High maturity; **(d)** Target is obscured.

To evaluate the model’s performance in any scenario, we utilized images of strawberries with varying degrees of ripeness as a test dataset. **Figures 9a–c** display the recognition performance of the model on strawberries of low, medium, and high maturity, respectively. These results indicate that the model can accurately identify strawberries at different stages of ripening and recognize the entire ripening process.

During the picking process of the robot, the uncrewed vehicle moved underneath, as captured by the camera on the robot arm and car. Owing to the complex structure of strawberries, which belong to the rose family of perennial herbs, stem roots can be divided into new stems, rhizomes, and stolons. Strawberry leaves are the base of three compound leaves with long petioles, which may cause the target object to be blocked by stems and leaves. Therefore, we selected occluded images to verify the model’s resistance to occlusions. **Figure 9d** shows that a low-ripening strawberry is occluded by the leaves of high-ripening strawberries, with leaf color similar to that of low-ripening strawberries. The recognition results demonstrated the model’s ability to accurately identify low-grade ripe strawberries, proving its suitability for real-world applications.

**Figure 10:**
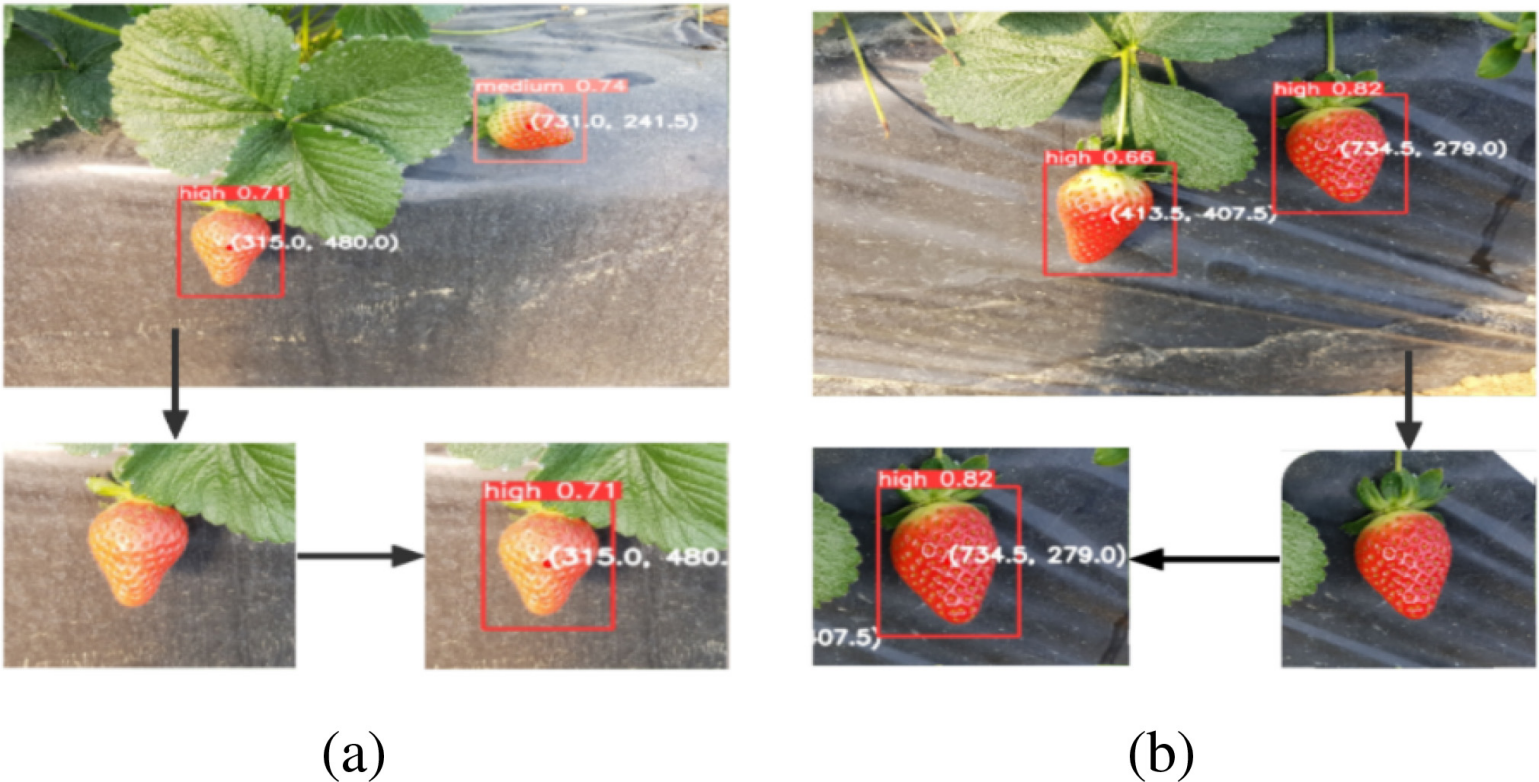
Different light conditions. **(a)** Environment with direct sunlight; **(b)** Environment with natural light.

Images of illuminated and gloomy strawberries were collected to test the model’s effectiveness under varying lighting conditions. In **Figure 10**, the left strawberry is exposed to sunlight, whereas the right strawberry is in the shadow. The recognition results demonstrate that the model performs well under both lighting conditions and is suitable for practical applications.

### 4.2 Ablation experiment

**Figure 11:**
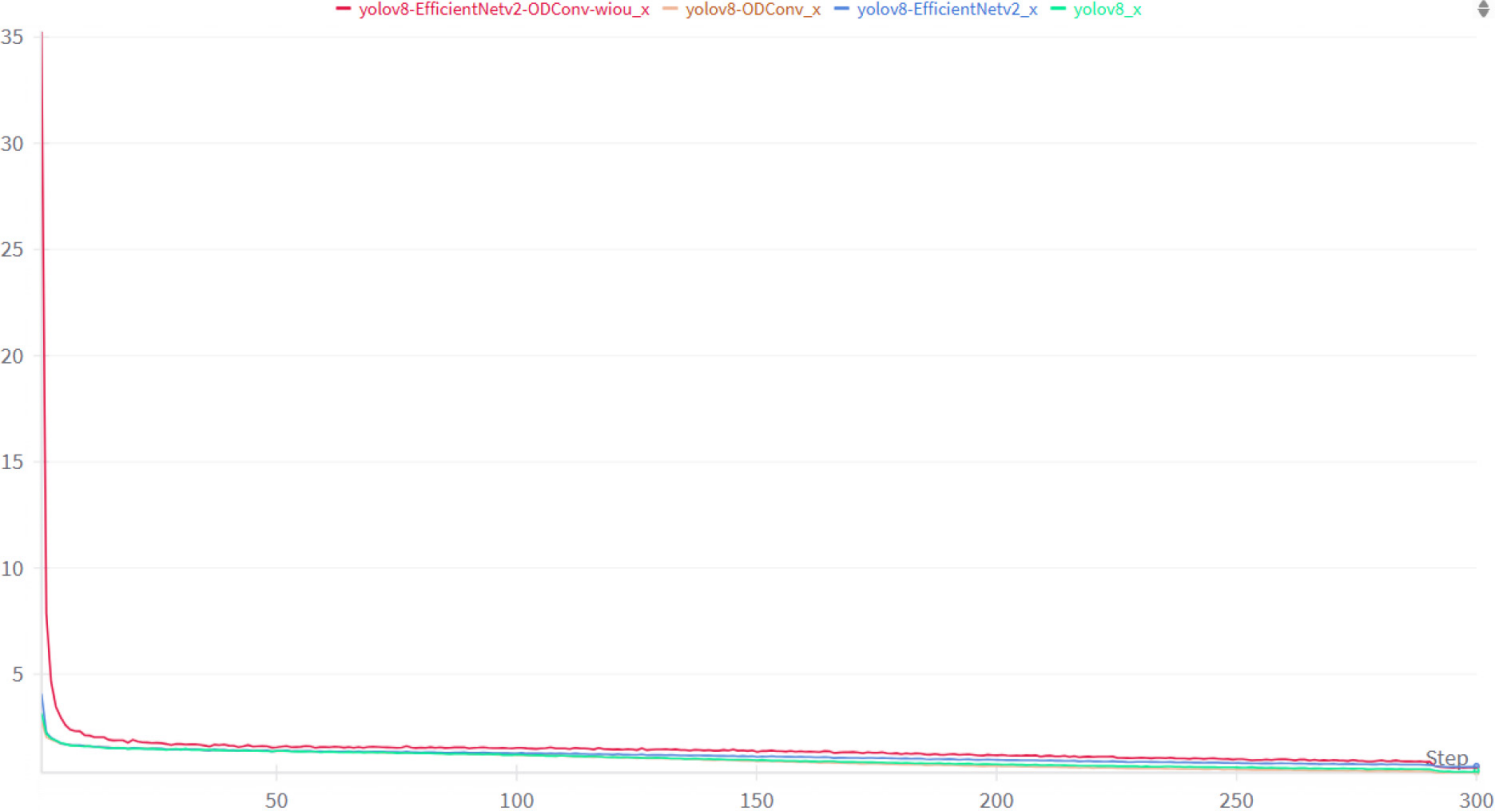
Training loss curve.

To verify the effectiveness of the module in the original YOLOv8 model, the same dataset and test set were used to conduct ablation experiments on the corresponding models. The performance parameters of each model are listed in **Table 2**. YOLOv8 represents the original YOLOv8 model, YOLOv8 + ODConv represents the model that replaces the standard convolution with a full-dimensional dynamic convolution at the neck, and YOLOv8 + EfficientV2 represents the model-replaced backbone network by the EfficientV2 network. YOLOv8 + EfficientV2 + ODConv indicates that the backbone network was replaced by the EfficientV2 network, while the neck part was modified, and the standard convolutions were replaced with ODConv. Simultaneously, the neck structure is changed, and the original ordinary convolution of the neck is replaced by a full-dimensional dynamic convolution. YOLOv8 + EfficientV2 + ODConv + WIoU represents a change from CIoU to WIoU based on previous improvements. The training loss function is shown in **Figure 11**. The YOLOv8-EfficientNetv2-ODConv-WIoU-x model converges faster, and the loss function decreases more rapidly and eventually stabilizes at a lower value. The performance at the end of training was relatively better.

**Table 2:** Ablation experiment.

Algorithm	IoU	Precision (%)	Recall (%)	mAP50	Parameter (M)	Speed/ms
YOLOv8	0.7	79.02	79.36	79.63	68.16	7.73
YOLOv8 + ODConv	0.7	87.92	85.44	91.75	68.71	8.142
YOLOv8 + EfficientV2	0.7	84.82	89.54	93.30	57.32	9.719
YOLOv8 + WiseIoU	0.7	81.6	83.93	85.75	68.16	6.4
YOLOv8 + EfficientV2 + ODConv	0.7	88.54	89.05	93.60	57.46	12.02
YOLOv8 + EfficientV2 + ODConv + WIoU	0.7	90.81	86.03	93.10	57.48	10.61

Because the model must be deployed on the picking robot, the number of parameters in the model must be minimized. The identified strawberries are divided into three types of maturity; therefore, it is necessary to accurately identify their maturity. Therefore, precision and parameter quantity are the key evaluation indexes. **Table 2** shows that the precision index improved significantly by 11.26% after adding full-dimensional dynamic convolution. However, the number of model parameters is too large; therefore, the EfficientV2 network is replaced with the original backbone network. The results show that the number of parameters is significantly reduced by 15.83% compared to the original YOLOv8 model. However, the precision improvement was not substantial. Therefore, the two models were combined. Compared to the original YOLOv8 model, the precision index of the new model was improved by 12.05%, and compared to the previous YOLOv8 + ODConv model, the precision index has been improved by 0.7%. At the same time, the number of parameters is only increased by 0.14 M compared with the YOLOv8 + EfficientV2 model. However, it is worth noting that the training reasoning time of this model was 4.29 ms slower than that of the original YOLOv8 model. To reduce the reasoning time and enhance the precision of identifying small targets, the original CIoU was replaced by the WIoU. The obtained data show that the precision index of the original YOLOv8 model continued to improve, which was 14.92% higher than that of the original YOLOV8 model. The training reasoning speed is 1.41 ms faster.

The experiments demonstrated that ODConv improved the precision index significantly, the EfficientV2 network effectively reduced the number of model parameters and their complexity, and WIoU accelerated the training reasoning speed and increased the recognition precision.

**Figure 12:**
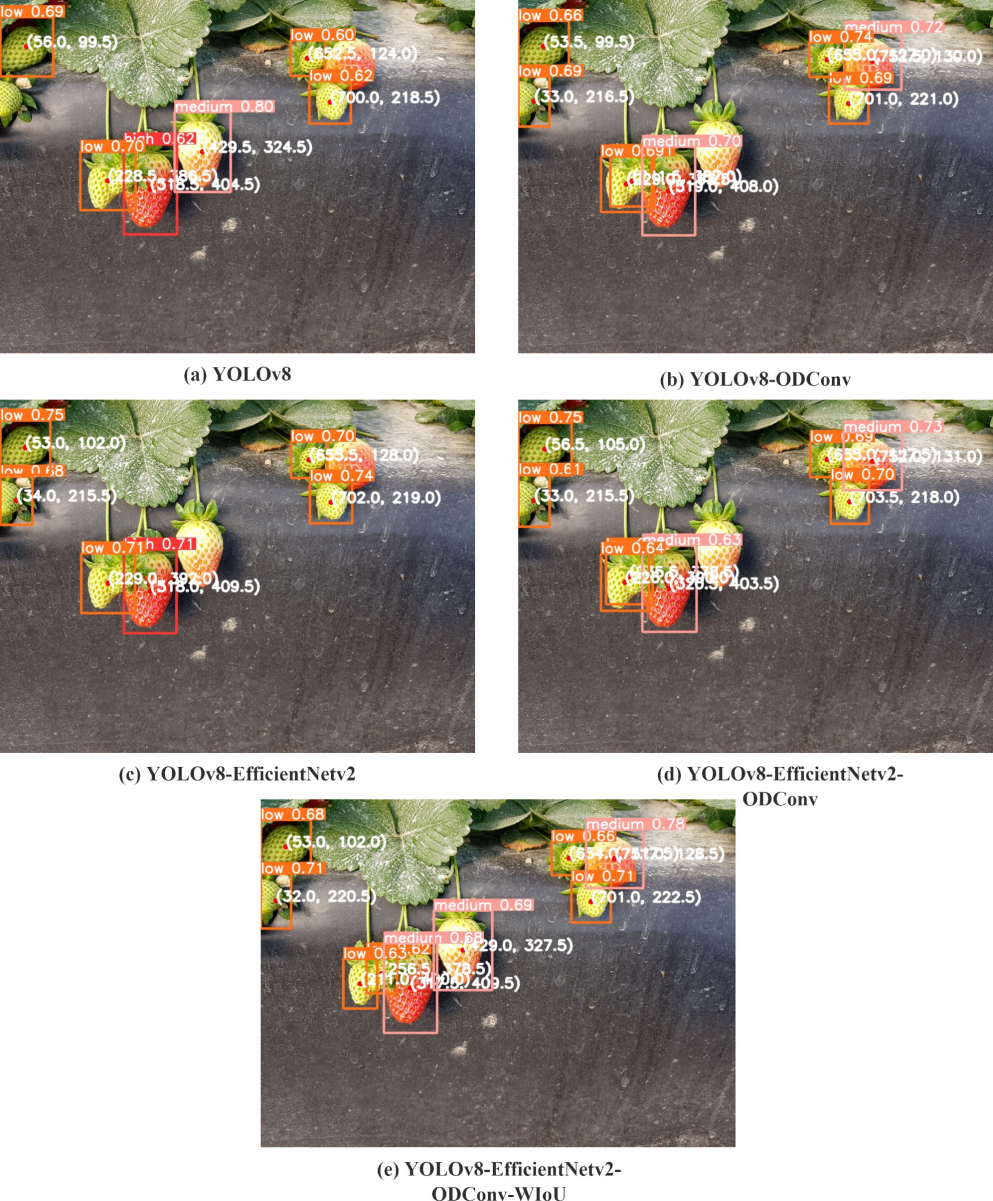
The results of different models. **(a)** YOLOv8; **(b)** YOLOv8-ODConv; **(c)** YOLOv8-EfficientNetv2; **(d)** YOLOv8-ODConv-EfficientNetv2; **(e)** YOLOv8-ODConv-EfficientNetv2-WIoU.

To compare the various models, we tested them using the same photograph, which included varying levels of maturity, lighting, and occlusion. **Figure 12a** illustrates the limitations of the original YOLOv8 algorithm in detecting low-ripe strawberries because it failed to identify a low-ripe strawberry on the left side and a small low-ripe strawberry in the middle. Additionally, it misclassifies medium-ripe strawberries as high-ripe strawberries and fails to detect medium-ripe strawberries on the right-hand side. As shown in **Figure 12b**, after adopting the full-dimensional dynamic convolution, strawberries with low maturity on the left and high maturity on the right can be identified, and tiny strawberries with low maturity in the middle can also be identified. owing to the small target size, the recognition box is slightly offset, and the strawberry initially identified as having high maturity can also be accurately identified as having medium maturity. However, strawberries that should have been identified as medium ripeness were not detected. As shown in **Figure 12c**, after the lightweight processing of the model using EfficientV2, the recognition effect is improved compared with the original YOLOv8 model. However, problems such as recognition errors and failure to identify small targets, have not improved. As shown in **Figure 12d**, when full-dimensional dynamic convolution and EfficientV2 are added simultaneously, the recognition effect is enhanced compared with the model with only EfficientV2. Small targets could also be identified, but the recognition box was still offset, and strawberries with middle and medium maturity were still not identified. As shown in **Figure 12e**, after replacing CIoU with WIoU, all strawberries could recognize the corresponding ripeness, and the small target recognition box offset was also improved.

Based on the experimental results, it can be concluded that the proposed model reduces complexity and improves recognition precision. The model accurately identifies strawberries in natural picking environments, making it suitable for practical application.

## 5 Discussion

To evaluate the effectiveness of different modules in the proposed model, we conducted ablation experiments using the same training and testing datasets and compared them with other similar modules. The performance metrics for each model are listed in **Table 3**.

**Table 3:** Comparison of the performance of different algorithm modules.

Algorithm	IoU	Precision (%)	Recall (%)	mAP50	Parameter (M)	Speed/ms
YOLOv8 + Vanillanet	0.7	79.32	89.05	90.45	38.21	2.86
YOLOv8 + EfficientV2	0.7	84.82	89.54	93.30	57.32	9.719
YOLOv8 + MobileOne	0.7	75.39	83.66	85.07	64.95	15.9
YOLOv8 + ODConv	0.7	87.92	85.44	91.75	68.71	8.142
YOLOv8 + DSConv	0.7	80.82	85.01	85.32	67.91	20.65
YOLOv8 + DWConv	0.7	84.18	80.96	85.21	64.47	5.92
YOLOv8 + EfficientV2 + ODConv + WIoU	0.7	90.81	86.03	93.10	57.48	10.61

As shown in **Table 3**, YOLOv8+EfficientV2 demonstrates a significant performance improvement, with an accuracy increase of 5.50%, recall improvement of 0.49%, and mAP50 increase of 2.85%. Although YOLOv8 + EfficientV2 has 50.03% more parameters than YOLOv8 + Vanillanet and is relatively slower, its improvements in accuracy and recall offer clear advantages. YOLOv8 + ODConv outperformed both YOLOv8 + DWConv and YOLOv8 + DSConv in terms of accuracy and recall, with a significant improvement in mAP50. Additionally, while the parameter count of YOLOv8 + ODConv is slightly higher than that of YOLOv8 + DWConv and YOLOv8 + DSConv, it delivers better performance and faster speed, outperforming YOLOv8 + DSConv by 20.65 ms and YOLOv8 + DWConv by 5.92 ms. YOLOv8 + EfficientV2 + ODConv + WIoU is a model with strong overall performance. This demonstrates a clear advantage over the models that incorporate only individual modules or networks.

To further validate the effectiveness of the proposed network, we conducted comparative experiments with several other object detection algorithms as well as different convolutional or network modules. All the models were trained using the same training dataset, and the training process consisted of 300 epochs.

As shown in **Table 4**, When comparing YOLOv3, YOLOv5, and YOLOv8, YOLOv5 performs the best in terms of accuracy, reaching 81.50%, which is 3.14% higher than YOLOv8 and 3.59% higher than YOLOv3. In terms of recall, YOLOv8’s recall rate of 79.36% is slightly higher than that of YOLOv5’s 76.53%. However, YOLOv8’s mAP50 is 79.63%, which was 0.08% higher than that of YOLOv5. Regarding speed, YOLOv3 was the fastest, with a processing time of 5.01 ms per image, 35.1% faster than YOLOv8, and 16.6% faster than YOLOv5.When comparing YOLOv8, SSD, and Faster R-CNN, YOLOv8 outperforms SSD in both accuracy and recall. However, Faster R-CNN achieved a higher accuracy, surpassing YOLOv8 by 4.48%, although it was relatively slower. Compared to SSD’s 5.02 ms, YOLOv8 was slower.

**Table 4:** Comparison of the performance of different object detection algorithms.

Algorithm	IoU	Precision (%)	Recall (%)	mAP50	Parameter (M)	Speed/ms
YOLOv8	0.7	79.02	79.36	79.63	68.16	7.73
YOLOv5	0.7	81.50	76.53	79.57	97.20	6.01
YOLOv3	0.7	78.41	78.83	79.26	103.96	5.01
SSD	0.7	74.6	57.6	68.2	47.12	5.02
Faste R-CNN	0.7	82.5	84.6	86.6	106	14.35
YOLOv8 + EfficientV2 + ODConv + WIoU	0.7	90.81	86.03	93.10	57.48	10.61

In the comparison between YOLOv8 + EfficientV2 + ODConv + WIoU, Faster R-CNN, and SSD, YOLOv8 + EfficientV2 + ODConv + WIoU achieved an accuracy of 90.81%, which was 8.31% and 16.21% higher than those of Faster R-CNN and SSD, respectively. Its recall rate of 86.03% and mAP50 of 93.10% far exceeded toes of both Faster R-CNN and SSD. Although the processing speed of YOLOv8 + EfficientV2 + ODConv + WIoU is slower than that of SSD, it has 45.76% fewer parameters than Faster R-CNN, demonstrating superior computational efficiency. Therefore, YOLOv8 + EfficientV2 + ODConv + WIoU has a significant advantage in applications requiring high accuracy and is well suited for scenarios that require high precision and lower computational load.

**Figure 13:**
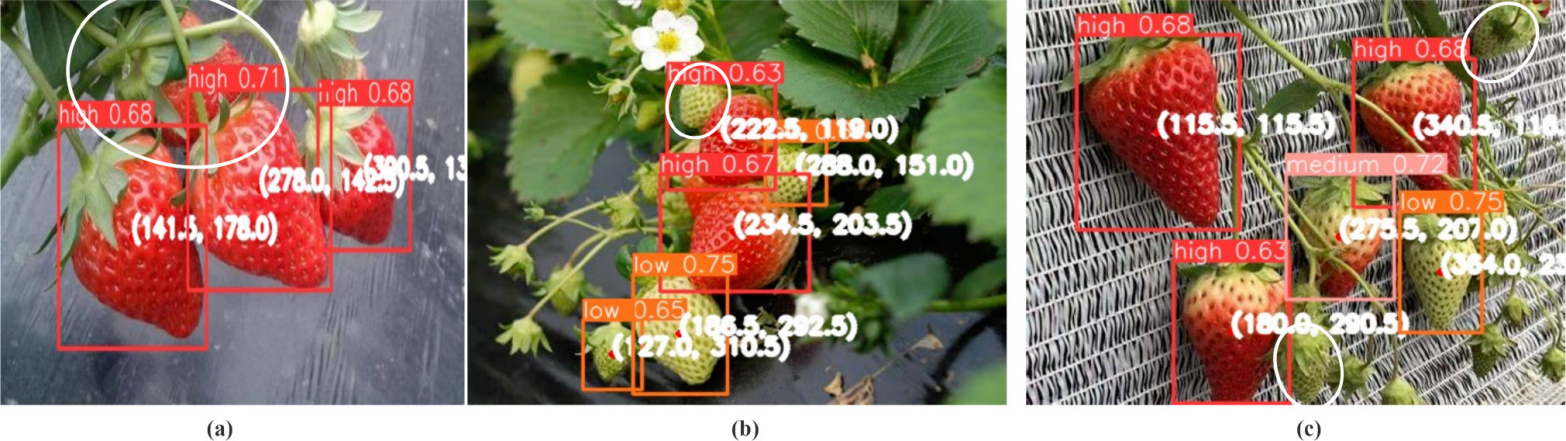
Detection results of Baidu PaddlePaddle dataset. **(a)** High maturity detection; **(b)** High and low maturity detection; **(c)** Three types of maturity detection.

To further validate the detection capability of the improved model, we conducted experiments using the Baidu Paddle-Paddle dataset. The detection results are shown in **Figures 13a–c**. In **Figure 13a**, the model can detect strawberries with high maturity, but one strawberry is blocked by the strawberries and leaves in front, and thus, is not detected. As shown in **Figure 13b**, most of the high and low-maturity strawberries were detected, but one low-maturity strawberry was missed. In **Figure 13c**, high-, medium-, and low-maturity strawberries were detected, but two low-maturity strawberries were not recognized due to interference from the top leaves. Therefore, the model may experience detection failures when the target object is extremely small or overly occluded by similar objects.

## 6 Conclusion

The target detection algorithm for strawberry-picking robots must overcome challenges such as occlusion, variations in lighting, and diversity in fruit shapes. To address these issues, this study proposes an improved YOLOv8 model that incorporates full-dimensional dynamic convolution, progressive learning strategies, and feature enhancement methods. The following conclusions were drawn based on validation using a strawberry dataset:

1. In this study, the new backbone architecture was 15.67% lighter than the original model to some extent. The addition of ODConv and the improvement of the neck structure improved the performance of the models, such as precision and recall.
2. The model can accurately identify targets in various picking environments, even when the target is blocked and the features of the target and occluder are similar. It achieves a precision of 90.81% with 57.48 M model parameters and is suitable for actual picking tasks and adaptability to changing light conditions, while maintaining effective identification performance.
3. The new model proposed in this study effectively identified strawberries at different stages of maturity in complex environments. However, there is room for improvement in the model’s computation speed. Future work will focus on enhancing the speed by integrating model optimization and hardware acceleration techniques with the current model, aiming to further improve the efficiency of mechanical harvesting. For instance, the model can be converted to the ONNX format and deployed on Jetson NX to generate a TensorRT-based inference engine, and Triton can be utilized for subsequent deployment.

## Data Availability

The raw data supporting the conclusions of this article will be made available by the authors, without undue reservation.
